# How Binding Affinity and Binding Specificity Map to Sequence Space

**DOI:** 10.1007/s00239-026-10308-5

**Published:** 2026-03-30

**Authors:** David A. Liberles, Michelle M. Meyer

**Affiliations:** 1 Department of Biology and Center for Computational Genetics and Genomics, Temple University, Philadelphia, PA 19122 USA; 2 Department of Biology, Boston College, Chestnut Hill, MA 02467 USA

**Keywords:** Binding affinity, Binding specificity, Protein interaction, RNA interaction, Protein folding, Aptamer

## Abstract

Evidence suggests that molecular interactions may evolve differently for proteins compared to functional RNA. We suggest that for RNA interacting with other nucleic acid molecules, the selection of higher affinity interactions leads to greater specificity, whereas for proteins interacting with other proteins, there is a trade-off between selection for greater affinity vs. greater specificity. This difference arises from the nature of the molecular contacts driving intra- and inter-molecular interactions, with crucial roles for non-specific hydrophobic interactions driving affinity in proteins and for specific hydrogen bonds driving affinity and specificity in RNA. The implications of this difference are discussed.

## Introduction

Genomes consist of thousands of protein-coding and RNA-expressing genes that function by interacting with other proteins, nucleic acids, and small molecules that are present in cells. These sets of interactions largely define the functions that make up the molecular phenotypes associated with a given species. For example, databases like STRING (Szklarczyk et al. 2023) and MINT (Calderone et al. 2020) detail the sets of protein–protein interactions that have either been observed biochemically or predicted in the proteomes of different species. Many proteins (both enzymes and non-enzymes) are part of metabolic and signal transduction pathways that function to produce either a yield of a particular metabolite or the generation of a transcriptional signal in the nucleus of a (eukaryotic) cell. These would be the functional interactions of a protein in a particular species, but would not reflect the totality of protein–protein interactions observed for any particular protein, as proteins not present in the same sub-cellular location at the same time will not functionally interact. A similar paradigm is true for RNAs. However, in the case of RNA-RNA interactions such contacts most frequently result in gene expression changes. The combination of all these interactions, each of which may be modified via mutation of the genome, define an organism that is under selection during evolution.

Current understanding of the evolution of genome architecture suggests that organismal effective population size is a critical modulator of selective strength (Lynch 2020). It describes the ability of a species to identify a relatively optimal sequence for any given selectable function (Liberles et al. 2011; Liberles 2023a). But the ability to find a suitably functional sequence for a given binding function also depends upon the underlying nature of the interactions themselves that are being selected (Buda et al. 2023). This is also a core part of the landscape and one that is much less well characterized in the evolutionary literature. This piece aims to offer a conceptual characterization of this, one that potentially uncovers core differences in the selectable nature of interactions of proteins and nucleic acids.

### Defining Sequence Space and the Relationship to Biochemical Functions

For any of the thousands of proteins in a genome, the number of possible sequences is 20^protein length, where the protein length is itself not fixed. For an RNA expressing gene, that set of possible sequences is 4^RNA length, where again the RNA length is not itself fixed. That is a large number of possible sequences, a subset of which will be sampled throughout evolution. Of all of the possible sequences for any biological macromolecule, a tiny fraction will have binding capabilities that correspond to interactions that are positively selected. It is occupation of this tiny part of possible sequence space that then enables a biomacromolecule to have a specific suite of binding interactions (Goldstein 2008; Liberles 2023a, b; Liberles et al. 2011). The sequences that occupy this space and emerge through selection will be largely distinct from those that are not positively selected (Goldstein 2008; Liberles et al. 2011). The chemical nature of the selected sequences is what defines their properties qualitatively.

### Defining Affinity and Specificity

A particular focal biomacromolecule will have an ensemble of interactions under physiological conditions at the concentration at which it is expressed (Dill and Bromberg 2010). More generally, it will have a potential affinity (binding energy) with every other sequence in sequence space, including the subset of sequences that are expressed in any given cell, as well as any small molecule metabolites present in the cellular milieu. The combination of binding energies and concentrations make most of the potential interactions for expressed molecules irrelevant, interactions that will rarely happen and not in sufficient mass numbers to have biological consequences. The relative binding energy dictates the relative fractional occupancy of two molecules about each other and the statistical average of those interactions for large numbers of individual molecules describes the concentration of different macromolecular complexes (Dill and Bromberg 2010). To add complexity to the scenario, any sequence can fold into an ensemble of structures, each with a distinct set of affinities (Siltberg-Liberles et al. 2011; Halvorsen et al 2010).

For the set of interactions of a particular sequence, mutations that alter the sequence may potentially change the binding affinities for all of the interactions (Dasmeh et al. 2024). Thus, the ensemble of potential interactions will change with mutations and is subject to selection. For any RNA or protein sequence with a particular expression level and resulting concentration, there is a set of total physical interactions with other biomolecules. However, within the total set of such interactions, there is a subset that are under positive or negative selection, and these would the set of interactions that occur under selection. Thus, we draw a distinction between physical interaction alone and evolutionary selection for that physical interaction.. In some cases, there will be a single interaction that is most strongly positively selected and the other interactions will be a byproduct of the resulting sequence and its physical properties. The distinction between selectable function and function that does not impact organismal fitness is a side issue to the arguments being made here, but is an important differentiation as interactions that are evolutionarily stable but not selected will persist without benefit to the organism solely because of the combination of mutational density in the sequences that enable the interaction. There is a now extensive literature on the importance of selection in defining function (see Doolittle et al. 2014 and references therein).

### Thinking About not Binding as a Kind of Binding

Selection to bind to a particular molecule restricts the space of available sequences, as many potential sequences will not have the ability to bind with sufficiently high affinity to the target molecule under physiological concentration and other conditions. Similarly, selection to not bind a particular molecule can also restrict the number of sequences to include only those that lack the ability to interact specifically with a particular target molecule (Liberles et al. 2011). One important question then becomes if the sequences that are excluded by the selective criteria for binding at a particular affinity overlap with the sequences that are excluded by the selective criteria for not binding other partners with a particular affinity. That is, what is the relationship between affinity and specificity? Does selecting for the highest affinity molecules automatically select against binding to other molecules or does selecting for the highest affinity molecules tend to select for molecules that have high affinity for other interactions as well?

### How Molecular Recognition Occurs in Proteins and in Nucleic Acids

Before addressing that question, let’s dive in to the nature of molecular recognition, of interactions that occur in proteins and in nucleic acids. In proteins, there isn’t a readily generalizable fit between interacting molecules. Hydrogen bonds in secondary structural units come from the backbone and not from amino acid side chains that are subject to mutation (Branden and Tooze 1999). Built from building blocks of amino acids, van der Waals interactions with steric fit between closely packed amino acids don’t follow a specific rule for how particular amino acids interact (Ames et al. 2016). Similarly, the interaction of charged amino acids follows the rules of Debye-Hückel theory, where the distance dependence depends upon the dielectric constant of the local environment, but does not have a regularity to orientations and distances. These interactions are stronger in more hydrophobic environments like binding interfaces where there is less charge screening. A key driving force is the hydrophobic effect, where the occlusion of water from tightly packing amino acids with relative hydrophobicity drives affinity (Ames et al. 2016). Because of the lack of regularity, any number of amino acids in the right orientation can pack together to occlude water. Aromatic residues do form very geometrically specific irregular interactions beyond the role associated with their hydrophobicity, sometimes interacting with each other, but these interactions contribute only a small fraction of the total binding affinity for protein–protein interactions in general (Anjana et al. 2012; Makwana and Mahalakshmi, 2015). The most specific interaction among aromatic residues was an edge to face interaction between Phe residues (Anjana et al. 2012). With that said, that some specific interactions (also including cation-pi interactions) occur that contribute to the binding energy specifically, these types of interactions do not drive the majority of the affinity in protein–protein interactions. Further, aromatic residues offer on average, statistically favorable interactions with ~ 40–50% of other residues according to statistical contact potentials (Miyazawa and Jernigan 1985).

For nucleic acids by contrast, every nucleobase in the chain can interact favorably with any other base, and ultimately folding is driven by pi-pi stacking of the hydrophobic bases to leave the charged edges of the planar base exposed to the solvent (Vicens and Kieft 2022). While canonical Watson–Crick base-pairing is fundamentally energetically driven by this stacking, the regularized hydrogen-bonding patterns that arise within the canonical double-stranded structure give rise to highly specific and high affinity intra- or inter-molecular interactions between two strands. However, Watson–Crick interactions are only a subset of the potential edge-edge interactions. The notion of sequence specific interactions can be extended to include the Hoogsteen edge of the base that remains exposed in the major groove of a Watson–Crick helix. This face enables regularized and reverse Hoogsteen base-pairs (T(U)-AT(U), and C + -GC base pairs in the Hoogsteen sense and A-AT(U) and G-GC base pairs in the reverse Hoogsteen sense) that may result in triple or quadruple helices, albeit with different preferences in RNA compared with DNA (Rangadurai et al. 2018; Leontis and Westhof 2001). Yet these interactions are still largely dependent on the bases involved with specific base combinations resulting stable conformations, and non-conforming bases substantially destabilizing the structure.

RNA tertiary structures are typically scaffolded by Watson–Crick paired elements, with regions not involved in Watson–Crick interactions typically forming complex motifs that incorporate non-canonical interactions to maximize stacking as described above. Adding complexity to the sequence to functional relationship for RNA is the dynamism of RNA structure. The time to fold is much shorter than the time required for synthesis resulting in rapid interchange between structures with similar folding energies. Thus, the probability of a specific base pairing pattern within the ensemble of potential stable structures is frequently invoked to understand the biological behavior of RNAs (Vicens and Kieft 2022; Ganser et al 2019).

### Affinity-Specificity Trade-Offs in Proteins and Nucleic Acids

**Fig. 1:**
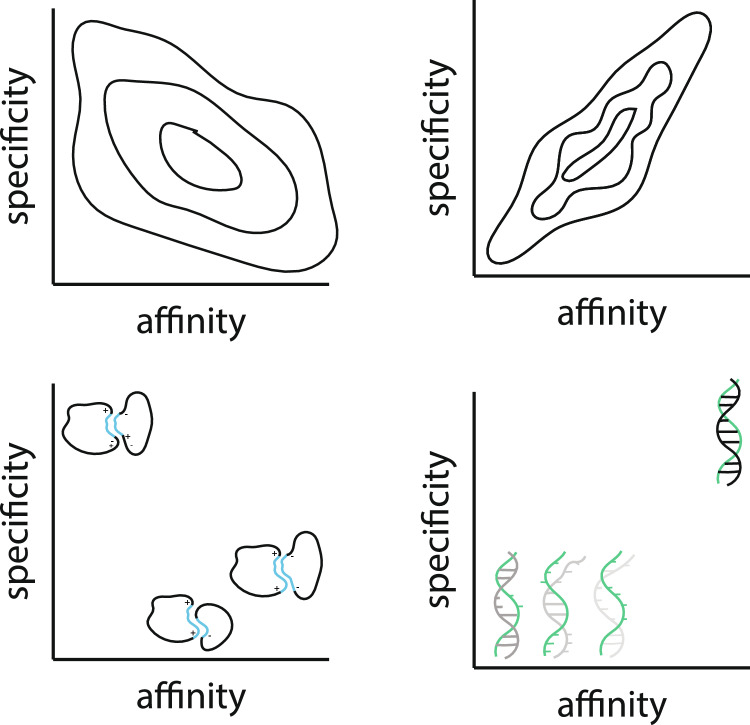
This schematic representation shows the different trade-offs between affinity and specificity between proteins and nucleic acids, with the affinity in one case driven by the hydrophobic surface areas, with some specificity coming from complementary charged interactions, and in the other case both affinity and specificity driven by base pairing (hydrogen bonding and coordinated stacking)

From the mechanisms giving rise to affinity, it is clear that there isn’t a general rule for specific amino acid-amino acid contacts that give rise to high affinity, specific interactions. The distribution of effects of mutations on interaction binding affinity shows a large variance, with many mutations having very small effects on a particular interaction when compared with wild type sequences (Ames et al. 2016; Brender and Zhang. 2015). The ad hoc nature of these interactions gives rise to a lack of specificity to the interactions that give rise to affinity. This is particularly true for large hydrophobic patches that give rise to affinity at binding interfaces (Desantis et al. 2022). While charges have been described as giving some specificity to protein–protein interactions as evidenced by the enrichment of charges in interfaces (Tsai et al. 1997), there are still likely to be many proteins that will have such charges, with residues of a particular charge representing 10–15% of possible amino acids at any site (this number comes from a sampling of the genetic code). The difference between matched and mismatched sequences is comparatively small, with high affinity driven by hydrophobic interactions and specificity driven by charge-charge interactions to a large extent. The highest affinity interactions will therefore have larger hydrophobic surface areas. From this, there is a natural tradeoff between affinity and specificity in protein–protein interactions. Evolving into sequences with higher affinity will more often result in sequences that are less specific in their interactions, with specificity generally harder to achieve in proteins than in nucleic acids. This is shown schematically in Fig. 1.

This contrasts with nucleic acids where the double-stranded structure characteristic of many biologically relevant intermolecular RNA-RNA or RNA–DNA interactions largely depends upon Watson–Crick interactions. While base stacking contributes significantly to the energetics, this contribution is dependent upon proper orientation that is steered through the hydrogen bonding interactions of base pairing (Delcourt and Blake 1991; Svozil et al. 2010). Examples of such base paired interactions include a host of regulatory interactions across all domains of life (siRNA, miRNA, sRNAs, etc.), tRNA-mRNA interactions that enable translation, interaction between the 16S rRNA and Shine-Dalgarno sequence that guide translation initiation in bacteria, and even CRISPR recognition of specific DNA or RNA sequences. The nature of Watson–Crick interactions enables both high affinity and highly specific interactions. One mark of the specificity with which such interactions act is the extent to which transcriptomes are under selection to prevent spurious regulatory RNA-RNA interactions (Umu et al. 2016; Hockenberry et al. 2018; Chen and Rajewsky 2006; Guo et al. 2008). The energetic effect of a single mismatched base in a stem structure is significant (Aboul-ela et al. 1985). The end effect of this is that a high affinity molecule is specific for a perfectly matched complement and sequences that are not perfectly matched are likely to exhibit an energetic penalty.

One caveat to note is that a cell operates at generally fixed temperature, pH, and salt/metal ion concentrations, as double-stranded structures grow longer, they gain in affinity, but in the environmental conditions will become less specific. However, there is a point that will optimize affinity and specificity for a given interaction in sequence space under cellular conditions. This has been observed in CRISPR guide RNA engineering efforts, where decreased affinity of the nucleic acid pairing ultimately increased specificity of the guided nuclease cleavage (Fu, 2014, Bisaria, 2017). From a theoretical perspective, one might expect that the length of stem loops is itself constrained by the organismal strength of selection. This is observed in many systems driven by mutation-selection-drift balance and is a well understood process with well understood effects on molecular stability (Wilson et al. 2020). In neither large nor small effective population size species would one expect to select for stem structures longer (and with greater affinity) than their selectable stability in cellular conditions. Therefore, under these conditions, one would expect RNA molecules to exist in a regime where stability and affinity are correlated, regardless of effective population size.

This argument generally holds with all macromolecular interactions that enable binding, including those that are subject to drift barriers, where the same evolutionary and biophysical dynamics interact around the added macromolecular complexity (Lynch 2020). There is an interplay between what is selected for based upon underlying thermodynamics, the strength of selection dependent upon organismal population size, and the collection of sequences available in sequence space. A similar argument as that for other macromolecular interactions,, can be made for some intrinsically disordered proteins. At least some intrinsically disordered proteins fold upon binding, where the energy of folding around the target (targets in the case of multi-specificity) is a mechanism of gaining specificity of interaction (Teilum et al. 2021). In this case, this entropic energetic cost in affinity is driving specificity. One recent study did suggest that disordered proteins can bind at high affinity and specificity, but driven by strong selection inducing strong sequence conservation (a reduction in the sampling of sequence space for key residues) (Lazar et al. 2022). Within the set of sequences, there is likely still a trade-off between affinity and specificity. Molecules with high affinity and specificity were not viewed in the context of sequence space and the density of solutions with variation in specificity and/or affinity.

An additional factor that is relevant to this discussion is macromolecular crowding in cellular milieus. The large concentration of molecules in cells inherently leads to more non-selected interactions occurring transiently and can also affect the affinity of selected interactions, both for ordered and for disordered proteins (Zosel et al. 2020). This itself will become part of the selective regime that sequences evolve in, but doesn’t alter the inherent biophysical tradeoffs between affinity and specificity in proteins and in nucleic acids.

Nucleic acids may also interact with a wide variety of molecules including proteins, metal ions, amino acids, and nucleoside analogs, in single-stranded, double-stranded or tertiary structured forms. Nucleic acid tertiary structures that interact specifically with small-molecules or proteins are termed aptamers, and while they can be generated synthetically via in vitro selection (Ellington and Szostak 1990; Tuerk and Gold 1990), they also occur naturally (Nahvi et al. 2002; Huang and Lilley 2025). It was originally argued that increased specificity in nucleic acid-small molecules interactions would result in increased affinity (Eaton 1995); however, experimental studies have found no specific relationship between affinity and specificity in large collections of synthetic aptamers (Carothers et al. 2006; Alkamis et al. 2025). While the properties of natural aptamers are distinct from those artificially selected (Kennedy et al. 2010; Meyers et al. 2004) there is no evidence to date that suggests a different paradigm to describe the relationship between affinity and specificity.

RNA–protein interactions also tend have no generalizable model of specific amino acids interacting with specific nucleotides. Even as datasets increase in size and complexity, computational prediction of RNA-RBP (RNA binding protein) interactions remains an active challenge in the field (Mizrahi et al 2025; Hentz et al. 2025) due to many factors, such as sequence accessibility and secondary or tertiary folding, that confound simple models for binding. Indeed, the relationship between primary sequence and functional conservation also remains obscure for many long noncoding RNAs (lncRNAs) as differing regions may act as isolated motifs (sequence or structural) that enable interaction with protein partners, and structural elements displaying Watson–Crick pairing scaffold these partners into close proximity (Fabbri et al. 2019). While there are a few general trends that have been noted DNA–protein interactions in the case of zinc finger transcription factor proteins (Klug 2010, see also Zhang et al. 2025), these are far from the 1:1 map of Watson–Crick base pairing that occur frequently between two nucleic acid molecules wherein specificity and affinity may correlate across an ensemble of interacting sequences.

## Concluding Thoughts

Given the inherent differences in molecular recognition between proteins and nucleic acids in self-recognition, the underlying sequence space differs in how it maps to functional, selected proteins and nucleic acids. This stems from the nature of the mapping in the spaces of protein–protein interactions vs. RNA-RNA interactions. In proteins, the lack of generalizable lock and key rules for how specific amino acids interact and the ubiquity of the hydrophobic effect in driving affinity leads to high affinity sequences that are not unique in their interactions. RNA, when self-interacting via Watson–Crick base-pairing, has a 1:1 mapping due to hydrogen bonding patterns. While there are many equivalent affinity interactions, these will all be specific interactions, and will evolve via coordinated (compensatory) changes in RNA molecules. The equivalent high affinity interactions in proteins will mostly occur through replacing less specific hydrophobic residues with other hydrophobic residues. When mutations occur for selection to act upon, there will be a general trade-off in finding sequences with higher affinity and those with higher specificity in proteins. In nucleic acid self-interaction, there will be a greater correlation between mutations that increase affinity and those that increase specificity. Thus, acquiring novel RNA-RNA interactions that are both high-affinity and high specificity may be relatively facile, leading to the plethora of RNA-RNA regulatory interactions observed across all species (Dexheimer and Cochella 2020; Dutcher and Raghavan 2018). This distinction is worth noting as researchers understand the evolution of protein and RNA sequences across genomes. It also is informative for what types of molecules are more likely to evolve promiscuous interactions (proteins), for when those interactions may open up new evolutionary trajectories, during adaptation, or during the early diversification of life.

## References

[CR1] Aboul-ela F, Koh D, Tinoco I Jr, Martin FH (1985) Base-base mismatches. Thermodynamics of double helix formation for dCA3XA3G + dCT3YT3G (X, Y = A,C,G,T). Nucleic Acids Res 13(13):4811–4824. 10.1093/nar/13.13.4811 4022774 10.1093/nar/13.13.4811 PMC321828

[CR2] Alkhamis O, Byrd C, Canoura J, Bacon A, Hill R, Xiao Y (2025) Exploring the relationship between aptamer binding thermodynamics, affinity, and specificity. Nucleic Acids Res 53(6):gkaf219. 10.1093/nar/gkaf219 40156861 10.1093/nar/gkaf219 PMC11952966

[CR3] Ames RM, Talavera D, Williams SG, Robertson DL, Lovell SC (2016) Binding interface change and cryptic variation in the evolution of protein-protein interactions. BMC Evol Biol 16:40. 10.1186/s12862-016-0608-1 26892785 10.1186/s12862-016-0608-1 PMC4758157

[CR4] Anjana R, Vaishnavi MK, Sherlin D, Kumar SP, Naveen K, Kanth PS, Sekar K (2012) Aromatic-aromatic interactions in structures of proteins and protein-DNA complexes: A study based on orientation and distance. Bioinformation 8:1220-4. 10.6026/97320630081220 23275723 10.6026/97320630081220 PMC3530875

[CR5] Bisaria N, Jarmoskaite I, Herschlag D (2017) Lessons from enzyme kinetics reveal specificity principles for RNA-guided nucleases in RNA interference and CRISPR-based genome editing. Cell Syst 4(1):21–29. 10.1016/j.cels.2016.12.010 28125791 10.1016/j.cels.2016.12.010 PMC5308874

[CR6] Branden CI, and Tooze J (1999) Introduction to Protein Structure (2nd ed.). Garland Science. 10.1201/9781136969898

[CR7] Brender JR, Zhang Y (2015) Predicting the effect of mutations on protein-protein binding interactions through structure-based interface profiles. PLoS Comput Biol 11(10):e1004494. 10.1371/journal.pcbi.1004494 26506533 10.1371/journal.pcbi.1004494 PMC4624718

[CR8] Buda K, Miton CM, Fan XC, Tokuriki N (2023) Molecular determinants of protein evolvability. Trends Biochem Sci 48(9):751–760. 10.1016/j.tibs.2023.05.009 37330341 10.1016/j.tibs.2023.05.009

[CR9] Calderone A, Iannuccelli M, Peluso D, Licata L (2020) Using the MINT Database to search protein interactions. Curr Protoc Bioinformatics 69(1):e93. 10.1002/cpbi.93 31945268 10.1002/cpbi.93

[CR10] Carothers JM, Oestreich SC, Szostak JW (2006) Aptamers selected for higher-affinity binding are not more specific for the target ligand. J Am Chem Soc 128(24):7929–7937. 10.1021/ja060952q 16771507 10.1021/ja060952q PMC4287982

[CR11] Chen K, Rajewsky N (2006) Natural selection on human microRNA binding sites inferred from SNP data. Nat Genet 38(12):1452–1456. 10.1038/ng1910 17072316 10.1038/ng1910

[CR12] Dasmeh P, Zheng J, Erdoğan AN, Tokuriki N, Wagner A (2024) Rapid evolutionary change in trait correlations of single proteins. Nat Commun 15(1):3327. 10.1038/s41467-024-46658-1 38637501 10.1038/s41467-024-46658-1 PMC11026499

[CR13] Delcourt SG, Blake RD (1991) Stacking energies in DNA. J Biol Chem 266(23):15160–15169 1869547

[CR14] Desantis F, Miotto M, Di Rienzo L et al (2022) Spatial organization of hydrophobic and charged residues affects protein thermal stability and binding affinity. Sci Rep 12:12087. 10.1038/s41598-022-16338-5 35840609 10.1038/s41598-022-16338-5 PMC9287411

[CR15] Dexheimer PJ, Cochella L (2020) MicroRNAs: from mechanism to organism. Front Cell Dev Biol 8:409. 10.3389/fcell.2020.00409 32582699 10.3389/fcell.2020.00409 PMC7283388

[CR16] Dill K, Bromberg S (2010) Molecular driving forces: Statistical thermodynamics in biology, chemistry, physics, and nanoscience, 2nd ed. Garland Science. 10.4324/9780203809075

[CR17] Doolittle WF, Brunet TD, Linquist S, Gregory TR (2014) Distinguishing between “function” and “effect” in genome biology. Genome Biol Evol 6(5):1234–1237. 10.1093/gbe/evu098 24814287 10.1093/gbe/evu098 PMC4041003

[CR18] Dutcher HA, Raghavan R (2018) Origin, evolution, and loss of bacterial small RNAs. Microbiol Spectr 6(2):10.1128/microbiolspec.rwr-0004-2017. 10.1128/microbiolspec.RWR-0004-2017 29623872 10.1128/microbiolspec.rwr-0004-2017 PMC5890949

[CR19] Eaton BE, Gold L, Zichi DA (1995) Let’s get specific: the relationship between specificity and affinity. Chem Biol 2(10):633–638. 10.1016/1074-5521(95)90023-3 9383468 10.1016/1074-5521(95)90023-3

[CR20] Ellington AD, Szostak JW (1990) In vitro selection of RNA molecules that bind specific ligands. Nature 346(6287):818–822. 10.1038/346818a0 1697402 10.1038/346818a0

[CR21] Fabbri M, Girnita L, Varani G, Calin GA (2019) Decrypting noncoding RNA interactions, structures, and functional networks. Genome Res 29(9):1377–1388. 10.1101/gr.247239.118 31434680 10.1101/gr.247239.118 PMC6724670

[CR22] Fu Y, Sander JD, Reyon D, Cascio VM, Joung JK (2014) Improving CRISPR-Cas nuclease specificity using truncated guide RNAs. Nat Biotechnol 32(3):279–284. 10.1038/nbt.808 24463574 10.1038/nbt.2808 PMC3988262

[CR23] Ganser LR, Kelly ML, Herschlag D, Al-Hashimi HM (2019) The roles of structural dynamics in the cellular functions of RNAs. Nat Rev Mol Cell Biol 20(8):474–489. 10.1038/s41580-019-0136-0 31182864 10.1038/s41580-019-0136-0 PMC7656661

[CR24] Goldstein RA (2008) The structure of protein evolution and the evolution of protein structure. Curr Opin Struct Biol 18(2):170–177. 10.1016/j.sbi.2008.01.006 18328690 10.1016/j.sbi.2008.01.006

[CR25] Guo X, Gui Y, Wang Y, Zhu QH, Helliwell C, Fan L (2008) Selection and mutation on microRNA target sequences during rice evolution. BMC Genomics 9:454. 10.1186/1471-2164-9-454 18831738 10.1186/1471-2164-9-454 PMC2567346

[CR26] Halvorsen M, Martin JS, Broadaway S, Laederach A (2010) Disease-associated mutations that alter the RNA structural ensemble. PLoS Genet 6(8):e1001074. 10.1371/journal.pgen.1001074 20808897 10.1371/journal.pgen.1001074 PMC2924325

[CR27] Hentze MW, Sommerkamp P, Ravi V, Gebauer F (2025) Rethinking RNA-binding proteins: Riboregulation challenges prevailing views. Cell 188(18):4811–4827. 10.1016/j.cell.2025.06.021 40912239 10.1016/j.cell.2025.06.021

[CR28] Hockenberry AJ, Jewett MC, Amaral LAN, Wilke CO (2018) Within-Gene Shine-Dalgarno Sequences Are Not Selected for Function. Mol Biol Evol 35(10):2487–2498. 10.1093/molbev/msy150 30085185 10.1093/molbev/msy150 PMC6188533

[CR29] Huang L, Lilley DMJ (2025) Some general principles of riboswitch structure and interactions with small-molecule ligands. Q Rev Biophys 58:e13. 10.1017/S0033583525100012 40432402 10.1017/S0033583525100012

[CR30] Kennedy R, Lladser ME, Wu Z, Zhang C, Yarus M, De Sterck H, Knight R (2010) Natural and artificial RNAs occupy the same restricted region of sequence space. RNA 16(2):280–289. 10.1261/rna.1923210 20032164 10.1261/rna.1923210 PMC2811657

[CR31] Klug A (2010) The discovery of zinc fingers and their development for practical applications in gene regulation and genome manipulation. Q Rev Biophys 43(1):1–21. 10.1017/S0033583510000089 20478078 10.1017/S0033583510000089

[CR32] Lazar T, Tantos A, Tompa P, Schad E (2022) Intrinsic protein disorder uncouples affinity from binding specificity. Protein Sci 31(11):e4455. 10.1002/pro.4455 36305763 10.1002/pro.4455 PMC9601785

[CR33] Leontis NB, Westhof E (2001) Geometric nomenclature and classification of RNA base pairs. RNA 7(4):499–512. 10.1017/s1355838201002515 11345429 10.1017/s1355838201002515 PMC1370104

[CR34] Liberles DA (2023a) The Memory Problem for Neutral Mutational Models of Evolution. J Mol Evol 91(1):2–5. 10.1007/s00239-022-10084-y 36562800 10.1007/s00239-022-10084-y

[CR35] Liberles DA (2023b) A Genomic Conceptualization of Species. J Mol Evol 91(4):379–381. 10.1007/s00239-023-10111-6 37079045 10.1007/s00239-023-10111-6

[CR36] Liberles DA, Tisdell MD, Grahnen JA (2011) Binding constraints on the evolution of enzymes and signalling proteins: The important role of negative pleiotropy. Proc Biol Sci 278(1714):1930–1935. 10.1098/rspb.2010.2637 21490020 10.1098/rspb.2010.2637 PMC3107659

[CR37] Lynch M (2020) The evolutionary scaling of cellular traits imposed by the drift barrier. Proc Natl Acad Sci U S A 117(19):10435–10444. 10.1073/pnas.2000446117 32345718 10.1073/pnas.2000446117 PMC7229682

[CR38] Makwana MK, Mahalakshmi R (2015) Implications of aromatic-aromatic interactions: From protein structures to peptide models. Protein Sci 24:1920–1933. 10.1002/pro.2814 26402741 10.1002/pro.2814 PMC4815235

[CR39] Meyers LA, Lee JF, Cowperthwaite M, Ellington AD (2004) The robustness of naturally and artificially selected nucleic acid secondary structures. J Mol Evol 58(6):681–691. 10.1007/s00239-004-2590-2 15461425 10.1007/s00239-004-2590-2

[CR40] Miyazawa S, Jernigan RL (1985) Estimation of effective interresidue contact energies from protein crystal-structures — Quasi-chemical approximation. Macromolecules 18:534–552

[CR41] Mizrahi O, Corley M, Feldman O, Fröhlking T, Sun L, Ziesel A, Antczak M, Bernetti M, Elhajjajy SI, Huang W, Nguyen GG, Park SS, Perez Martell RI, Trinity L, Xu K, Zok T, Bussi G, Jabbari H, Orenstein Y, Aviran S, Meyer MM, Yeo GW (2025) Evaluation of novel computational methods to identify RNA-binding protein footprints from structural data. RNA 31(8):1103–1124. 10.1261/rna.080215.124 40399037 10.1261/rna.080215.124 PMC12265944

[CR42] Nahvi A, Sudarsan N, Ebert MS, Zou X, Brown KL, Breaker RR (2002) Genetic control by a metabolite binding mRNA. Chem Biol 9(9):1043. 10.1016/s1074-5521(02)00224-7 12323379 10.1016/s1074-5521(02)00224-7

[CR43] Rangadurai A, Zhou H, Merriman DK, Meiser N, Liu B, Shi H, Szymanski ES, Al-Hashimi HM (2018) Why are Hoogsteen base pairs energetically disfavored in A-RNA compared to B-DNA? Nucleic Acids Res 46(20):11099–11114. 10.1093/nar/gky885 30285154 10.1093/nar/gky885 PMC6237737

[CR44] Siltberg-Liberles J, Grahnen JA, Liberles DA (2011) The evolution of protein structures and structural ensembles under functional constraint. Genes (Basel) 2(4):748–762. 10.3390/genes2040748 24710290 10.3390/genes2040748 PMC3927589

[CR45] Svozil D, Hobza P, Sponer J (2010) Comparison of intrinsic stacking energies of ten unique dinucleotide steps in A-RNA and B-DNA duplexes. Can we determine correct order of stability by quantum-chemical calculations? J Phys Chem B 114(2):1191–1203. 10.1021/jp910788e 20000584 10.1021/jp910788e

[CR46] Szklarczyk D, Kirsch R, Koutrouli M, Nastou K, Mehryary F, Hachilif R, Gable AL, Fang T, Doncheva NT, Pyysalo S, Bork P, Jensen LJ, von Mering C (2023) The STRING database in 2023: Protein-protein association networks and functional enrichment analyses for any sequenced genome of interest. Nucleic Acids Res 51(D1):D638–D646. 10.1093/nar/gkac1000 36370105 10.1093/nar/gkac1000 PMC9825434

[CR47] Teilum K, Olsen JG, Kragelund BB (2021) On the specificity of protein–protein interactions in the context of disorder. Biochem J 478:2035–2050. 10.1042/BCJ20200828 34101805 10.1042/BCJ20200828 PMC8203207

[CR48] Tsai CJ, Lin SL, Wolfson HJ, Nussinov R (1997) Studies of protein-protein interfaces: a statistical analysis of the hydrophobic effect. Protein Sci 6(1):53–64. 10.1002/pro.5560060106 9007976 10.1002/pro.5560060106 PMC2143524

[CR49] Tuerk C, Gold L (1990) Systematic evolution of ligands by exponential enrichment: RNA ligands to bacteriophage T4 DNA polymerase. Science 249(4968):505–510. 10.1126/science.2200121 2200121 10.1126/science.2200121

[CR50] Umu SU, Poole AM, Dobson RC, Gardner PP (2016) Avoidance of stochastic RNA interactions can be harnessed to control protein expression levels in bacteria and archaea. Elife 5:e13479. 10.7554/eLife.13479 27642845 10.7554/eLife.13479 PMC5028192

[CR51] Vicens Q, Kieft JS (2022) Thoughts on how to think (and talk) about RNA structure. Proc Natl Acad Sci U S A 119(17):e2112677119. 10.1073/pnas.2112677119 35439059 10.1073/pnas.2112677119 PMC9169933

[CR52] Wilson AE, Kosater WM, Liberles DA (2020) Evolutionary processes and biophysical mechanisms: revisiting why evolved proteins are marginally stable. J Mol Evol 88:415–417. 10.1007/s00239-020-09948-y 32385626 10.1007/s00239-020-09948-y

[CR53] Zhang Y, Silvernail I, Lin Z, Lin X (2025) Interpretable protein-DNA interactions captured by structure-sequence optimization. Elife 14:RP105565. 10.7554/eLife.105565 40673435 10.7554/eLife.105565 PMC12270484

[CR54] Zosel F, Soranno A, Buholzer KJ, Nettels D, Schuler B (2020) Depletion interactions modulate the binding between disordered proteins in crowded environments. Proc Natl Acad Sci U S A 117(24):13480–13489. 10.1073/pnas.1921617117 32487732 10.1073/pnas.1921617117 PMC7306994

